# A Year of War and Drug Prices

**Published:** 1915-08-07

**Authors:** 


### A YEAR OF WAR AND DRUG PRICES.

Hardly any branch of commerce has been so greatly influenced by the war as the drug market, and a comparison of the prices quoted for drugs at this date last year with those ruling at the present time, after the conflict has been going on for just a year, is not only interesting, but gives some indication of what the course of the market may be so long as the war continues. It is only natural to find that the medicinal products which have been affected most seriously are those which were formerly obtained almost exclusively from Germany.

The moment war was declared the main source of supply was cut off, and had it not been for small supplies which have arrived from time to time from neutral countries—notably Switzerland and the United States—the small stocks that were already in the country would soon have been exhausted, for British manufacturers have been unable to produce these drugs in large commercial quantities, one reason being that they have had to work at full pressure even to keep pace with the increased demand for their ordinary products. The three drugs that have advanced to the most remarkable extent are acetyl-salicylic acid, salicylic acid, and sodium salicylate. For the first two of these fifteen times the pre-war price now has to be paid, and the third is twelve times the price quoted at the beginning of August last year. while phenacetin and acetanilide are seven times the pre-war prices.

Until a year ago, of course, practically all our supplies of these products were obtained from Germany, and this applies to most of the synthetic chemicals used in medicine. But the rise in prices has been by no means confined to synthetic drugs, the war having affected a very wide range of products. To continue the comparison, cocaine, guaiacol carbonate, and hexamine are three times the price quoted before the war; phenazone, atropine, paraldehyde, and carbolic acid five times; salol is nine times the pre-war price; and potassium permanganate eight times. Many more examples could be given, but the above will serve to give some indication of the extraordinary influence of the war on the drug market. One looks in vain for any sign that the general advancing tendency of prices is likely to cease before the war is over.

Hospitals and similar institutions, being, of course, among the largest buyers of drugs, are seriously affected by the shortage, and it will only be by the encouragement of economical prescribing and the use of parallel drugs obtainable at reasonable prices that it will be possible to prevent a great increase in the drug bills. We need not allude again to the correspondence on this subject which has appeared in The Hospital, but the importance of intimate relations between the hospital pharmacists and the medical staffs was one of the main points insisted on in the writers' proposals for securing economy in the drug bill.

